# Onset of tics in an elderly patient undergoing androgen deprivation therapy for prostate cancer: a case report

**DOI:** 10.1093/omcr/omae206

**Published:** 2025-03-20

**Authors:** Kim Madundo, Godwin Nnko, Leila Mwakipunda, Glory Makupa, Furaha Serventi

**Affiliations:** Department of Psychiatry and Mental Health, Kilimanjaro Christian Medical Centre, Sokoine Road, Moshi Box 3010, Tanzania; Department of Psychiatry and Mental Health, Kilimanjaro Christian Medical University College, Sokoine Road, Moshi Box 2240, Tanzania; Department of Oncology, Kilimanjaro Christian Medical Centre, Sokoine Road, Moshi Box 3010, Tanzania; Department of Oncology, Kilimanjaro Christian Medical Centre, Sokoine Road, Moshi Box 3010, Tanzania; Department of Oncology, Kilimanjaro Christian Medical Centre, Sokoine Road, Moshi Box 3010, Tanzania; Department of Oncology, Kilimanjaro Christian Medical University College, Sokoine Road, Moshi Box 2240, Tanzania; Department of Oncology, Kilimanjaro Christian Medical Centre, Sokoine Road, Moshi Box 3010, Tanzania; Department of Oncology, Kilimanjaro Christian Medical University College, Sokoine Road, Moshi Box 2240, Tanzania

**Keywords:** tics, tic disorder, androgen deprivation, prostate cancer, adverse effect

## Abstract

Motor and vocal tics typically manifest during childhood. Less often, tics can occur due to the use of substances or medications which raise dopamine levels. This case report describes an unusual occurrence of tics in an elderly man undergoing androgen deprivation with Goserelin for prostate cancer. Comprehensive assessments ruled out infectious, metabolic, psychiatric, and neurological conditions. The tics resolved with low-dose Risperidone, without adverse effects. This case underscores the potential for medication-induced tics in older adults, highlighting the need for increased awareness among healthcare providers regarding the neuropsychiatric effects of cancer therapies. Future research should further investigate these phenomena and their implications for treatment strategies.

## Introduction

Tics are involuntary, sudden, repetitive but non-rhythmic movements or sounds that are often difficult to control. Motor tics frequently present as quick eye blinking, rapid movements of the tongue or mouth, or twitching of the shoulder or neck. Vocal tics include humming, hissing, clearing the throat, barking, or repeating one’s sounds (echolalia). Tics predominantly begin during childhood, with average age of onset between 4 and 6. They are not associated with performing specific tasks but can worsen under emotional distress [1].

There are genetic and non-genetic aetiologies of tics. Previous literature has associated single dopamine-specific gene abnormalities with tic disorders [2]. Non-genetic causes include infectious agents (streptococcus bacteria) [3], lesions of the basal ganglia [4], and, less commonly, substances that increase central dopamine levels [1, 5]. Medications, including chemotherapeutic agents, can target and affect the neuroendocrine system. Goserelin, a synthetic analogue of Gonadotrophin-releasing hormone has been used to treat prostate cancer through androgen deprivation [6] but inadvertently raises central levels of monoamines including dopamine [7]. Other involuntary movements, such as chorea, have also been associated with use of gonadotropin analogues [6].

Tic disorders are diagnosed based on clinical presentation, and by ruling out comorbid medical conditions or substance use [1]. Neuroimaging methods such as functional MRI have been used for diagnostic classification in high-resource clinical research settings [8].

This study reports an uncommon onset of tics in an elderly patient receiving hormonal therapy for prostate cancer in a resource-limited setting.

## Case report

A man in his 80s presented with intensifying twitching of his mouth and tongue over several months. He experienced difficulty in refraining from humming and clearing his throat, particularly before falling asleep at night. These movements and vocalizations led to self-consciousness and interfered with his and his wife’s sleep. He also noticed recent grinding of his teeth and periodic restlessness. He denied experiencing neurological symptoms such as loss of consciousness, headaches, dizziness, or convulsions.

A year ago, he was diagnosed with low-grade invasive adenocarcinoma of the prostate (Gleason score 6). He has since been treated with androgen deprivation therapy: injections Goserelin 10.8 mg every three months and tablets Bicalutamide 50 mg daily. He also receives supplementation with tablets Calcium carbonate 500 mg daily and tablets Vitamin D 200 IU daily to prevent bone loss associated with androgen deprivation. He had no other chronic health conditions.

General examination was unremarkable. He was alert, walked slowly supporting himself with a walking stick, but otherwise well-coordinated and balanced. He exhibited abrupt and repetitive movements of his lips, tongue, and shoulders. The movements each lasted less than a second in duration and occurred in irregular bursts of repetitions. Less often, he hummed repetitively particularly when discussing sensitive matters such as decreased sexual functioning and adjusting to retirement. He had no other abnormal physical examination findings.

On mental status examination, he appeared well-dressed, cooperative, and friendly. He spoke coherently, with normal thought content and flow. He reported a slightly anxious mood while first attending the mental health clinic, but otherwise expressed a normal range of emotions. He scored 26/30 on the Montreal Cognitive Assessment indicating normal cognitive functioning. Considering the mild anxiety and interferences with sleep, screeners were conducted to rule out generalized anxiety (GAD7 score = 5/21) and depression (PHQ9 score = 4/27).

Investigations revealed a borderline low erythrocyte level (4.52 × 10^12^/L) but otherwise normal blood count. His erythrocyte sedimentation rate (5 mm/hr.), electrolytes (sodium, potassium, calcium, chlorine, magnesium), blood glucose, prolactin, cardiac, liver, and renal functions were also normal. He had castrate levels of testosterone (4.6 ng/ml) and a normal prostate-specific antigen (0.162 ng/ml). CT scan of the spine revealed marginal osteophyte formation at the L4/5 level and a symmetrical broad disc bulge at L3 to S1 levels, with no signs of metastases.

Considering the motor and vocal tics, associated bruxism and restlessness, the timing of onset, and the absence of other significant neurological or psychological phenomena, a diagnosis of ‘Other specified tic disorder, with onset after use of Goserelin’ was made.

The patient was prescribed tablets Risperidone 0.5 mg daily at night, for its antagonistic effect on dopamine [9]. He was educated about the likely cause of his symptoms and the chance of adverse effects. After one week, he was reviewed and reported a significant reduction of both motor and vocal tics. He did not experience adverse effects associated with neuroleptic use. The neuroleptics were continued at the same dose.

## Discussion

This report highlights an unusual onset of tics in an elderly patient following the administration of Goserelin. While tics typically manifest during childhood, this case underscores the potential for tics to emerge during old age, especially with pharmacotherapy use. The patient’s brief and rapid involuntary movements and vocalizations, alongside normal cognition and absence of neurological symptoms, support a diagnosis of tics related to Goserelin use.

The likely underlying pathophysiology is increased central dopamine levels, which have been implicated in tic disorders [2, 4, 5]. Symptoms including bruxism, chorea, and restlessness are commonly associated with excess dopamine [6, 10]. Furthermore, the absence of significant comorbidities and normal neurological examination reinforces the likelihood that the tics were medication-induced rather than from other pathologies.

The present case demonstrated a favourable outcome with low-dose Risperidone, a dopamine antagonist. The limitations of this case include potential confounders, such as concurrent medication use and short-term follow-up. Additionally, the subjective nature of tic severity assessment and the absence of standardized scales limit the generalizability of the findings. Functional brain imaging and autoimmune assays would be useful for diagnostics but were unavailable due to resource limitations.

In conclusion, this report emphasizes the need for increased awareness among healthcare providers regarding the potential for medication-induced tics in adult patients. Future research should focus on elucidating other neuropsychiatric effects of cancer treatment and exploring their safety and efficacy.
